# Cutaneous Granular Cell Tumor of the Breast: A Clinical Diagnostic Pitfall

**DOI:** 10.4021/jocmr403w

**Published:** 2010-08-18

**Authors:** Jose Aneiros-Fernandez, Salvador Arias-Santiago, Husein Husein-ElAhmed, Francisco OValle, Maria Ines Aroca Siendones, Jose Aneiros-Cachaza

**Affiliations:** a Department of Pathology, University Hospital, Granada, Spain; b Department of Dermatology, University Hospital, Granada, Spain; c Department Coordination of Tumor Bank Network Andalucia, San Cecilio University Hospital, Spain

## Abstract

We report the clinical-morphological study of a granular cell tumor in dermal/hypodermal junction and subcutaneous fat left breast of an 83-year-old woman with a family history of breast carcinoma. Mammography study showed a spiculated lesion in the lower inner quadrant with suspicion of malignancy. The results of fine needle puncture-aspiration were inconclusive. Subsequent tumorectomy revealed a poorly-defined indurated lesion of 1.1 × 0.7 cm. The histopathology study showed a proliferation of cells with ample and granular cytoplasm that were positive for S100, CD 68 and inhibin and negative for hormonal receptors. We present a benign lesion that clinically reproduces a breast carcinoma.

**Keywords:**

Granular cell tumor; Breast; Differential diagnosis; Cutaneous

## Introduction

Granular cell tumor (GCT) is a rare benign neoplasm of soft tissues of probable peripheral nervous tissue or Schwann cell origin. The most frequent site is the tongue (40% of cases), but it has been reported in various localizations (skin and visceral). In around 5% - 6% of cases GCT is observed in the breast [1], where it can be clinically and radiologically confused with a malignant breast tumor.

We report a case of GCT of the breast in an 83-year-old woman with clinical and radiology findings that mimic breast carcinoma, contributing clinical-morphological data on a lesion at an uncommon site.

## Case Report

A 83-year-old woman presented with an abnormality detected by screening mammography three months earlier. Her mother and sister had a history of breast cancer. Physical examination revealed a painless palpable mass of around 1 cm in the lower inner quadrant of the left breast, with no skin alteration or nipple discharge.

**Figure 1.**
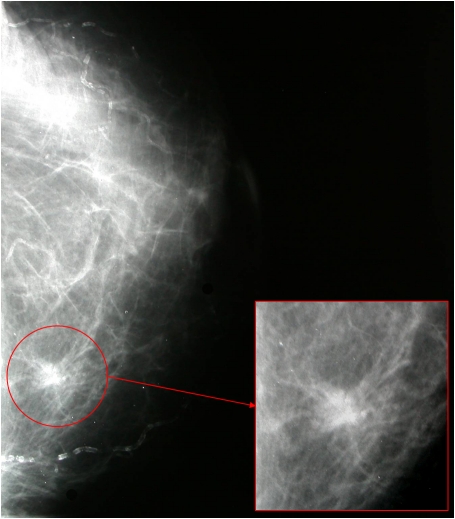
Mammography showed an ill-defined, high-density spiculated mass.

**Figure 2.**
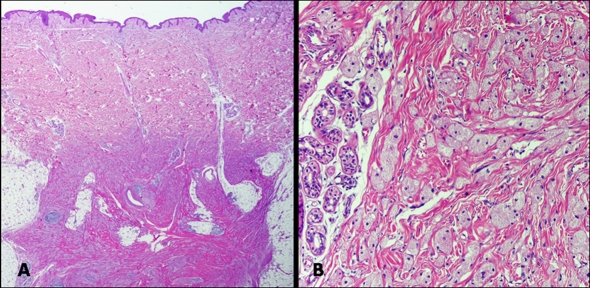
(A) Tumor ill-defined in dermal-hypodermal junction and subcutaneous fat. (Haematoxylin-eosin, original magnification: x 40); (B) Nests and sheets of cells containing eosinophilic cytoplasmic granules (haematoxylin-eosin, original magnification: x 200).

**Figure 3.**
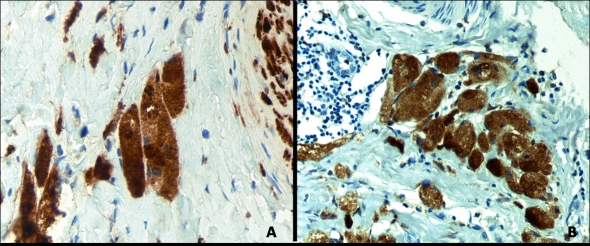
Positive staining of neoplastic cells for: (A) CD 68 (original magnification: x 400) and(B) S-100 protein (original magnification: x 400).

Mammography showed an ill-defined, high-density spiculated mass of 1 cm in left inner breast (Fig. 1). No associated calcifications were observed within the mass. Ultrasound revealed a poorly-defined, hypoechoic solid lesion disrupting fascial planes with a strong acoustic shadowing. We classified the mass as category 4c under the Breast Imaging Reporting and Data System (BI-RADS), i.e., with an estimated likelihood of malignancy of 50% - 90%. Results of fine needle aspiration cytology were inconclusive. Radioguided tumorectomy was performed due to clinical suspicion of malignant tumor, evidencing an ill-defined, spiculated, and indurated lesion of 1.1 × 0.7 cm. Histological examination showed an ill-defined firm nodule composed of compact nests and sheets of cells containing eosinophilic cytoplasmic granules with well-defined cell borders and prominent nucleoli (Fig. 2). This lesion was location in dermo-hypodermal junction and subcutaneous fat. The tumor cells were arranged in a fascicular pattern with an infiltrating growth pattern at the margins. The granules were PAS-positive and diastase-resistant in varying degrees of intensity. The immunohistochemical study showed that tumor cells were positive for S100 protein, vimentin, inhibin, and CD68 and were negative for cytokeratin AE1/AE3, smooth muscle actin, desmin, and estrogen and progesterone receptors (Fig. 3). Ki-67 revealed low nuclear proliferative activity. The tumor was completely excised. No evidence of tumor recurrence in left breast has been detected after four-years of follow-up.

## Discussion

Granular cell tumor was first described by Abrikossoft in 1926. It was originally designated myoblastoma [2] and considered to derive from smooth muscle, due to its localization in tongue and pharynx in the first reported cases. Subsequent studies using S100 staining and electron microscopy found that the tumor cells originate in Schwamm cells of peripheral nerves [3, 4] and can appear at any site (skin, lymph nodes, appendix, etc.).

The most frequent localizations are the head and neck (> 50% of cases) and the tongue (≈ 40% of cases). GCT of the breast is very uncommon, representing around 1/1,000 cases of breast cancer [1]. Analysis of data from the 25 cases reported in the literature and the present case (22 females and 3 males) revealed that premenopausal women were the most frequently affected, with a mean age at onset of 40.3 years (range: 17 - 83 years). We have found no report of GCT of breast in a woman as old as our 83-year-old patient. [1, 5-12]. The low reported incidence in males may be explained by their much less frequent participation in regular mammography screening. This tumor is most frequently observed in upper inner quadrant, whereas it was localized in the lower inner quadrant in our patient.

On radiographic or ultrasound images, GCT appears as a well-defined mass or as an infiltrating spiculated lesion, with no microcalcifications described in the majority of reports [6-12]. In the present case, it appeared as a poorly-defined infiltrating mass with clinical suspicion of malignity, as reported in 29% of the 24 cases reviewed.

The two reports of magnetic resonance images of GCT are controversial. One describes a homogenously enhanced mass on T1-weighted imaging with high signal intensity rim on T2-weighted sequence, and a high T2-signal has been shown to be a sign of benign disease [13]. The other describes a homogeneously enhanced mass with no true peripheral rim or peripheral predominance of T1 signal, which showed a very low signal on T2-weighted imaging, which is more frequently observed in malignant than benign lesions of the breast [14].

Microscopically, CGT is characterized by rounded, polygonal, and occasionally elongated cells with a wide and granular cytoplasm; the nuclei can be small and hyperchromatic or larger and vesicular. A similar morphology can be observed in smooth muscle tumors, malignant melanoma, angiosarcoma, and even breast carcinomas [15]. The diagnosis of CGT can be established by immunohistochemistry or by the demonstration of electrodense granular structures corresponding to secondary lisosomes.

Less than 1% of CGTs are malignant. The histopathologic criteria for malignity are: tumor more than 5 cm, presence of necrosis, high mitotic activity, and nuclear pleomorphism. These criteria were not met in the present case.

We highlight the association of CGT with infiltrating ductal carcinoma, which has been described in three patients of advanced age [16]. Usually CGT is treated by wide local excision to avoid local recurrences.

The message of the present report is that CGT of the breast should be included in the clinical differential diagnosis with carcinoma, especially in elderly patients with risk factors.
